# Where and how do young people like to get their sexual and reproductive health (SRH) information? Experiences from students in higher learning institutions in Mbeya, Tanzania: a cross-sectional study

**DOI:** 10.1186/s12889-021-11728-2

**Published:** 2021-09-16

**Authors:** Ruby Doryn Mcharo, Philippe Mayaud, Sia E. Msuya

**Affiliations:** 1 National Institute for Medical Research-Mbeya Medical Research Centre (NIMR-MMRC), Mbeya, Tanzania; 2 Department of Epidemiology & Biostatistics, Institute of Public Health, Kilimanjaro Christian Medical University College (KCMUCo), Moshi, Tanzania; 3 London School of Hygiene and Tropical Medicine (LSHTM), London, UK; 4 Department of Community Health, Institute of Public Health, Kilimanjaro Christian Medical University College (KCMUCo), Moshi, Tanzania; 5 Community Health Department, Kilimanjaro Christian Medical Centre (KCMC), Moshi, Tanzania

**Keywords:** Sexual and reproductive health (SRH), Parent-child communication, Young adults, Adolescents, Sexual behavior, Tanzania

## Abstract

**Background:**

Sexual and reproductive health (SRH) among young adults in low- and middle-income countries (LMIC) is still a major public health challenge. Early school-based sexuality education programs and sexual health information sharing between teachers, parents and young people have been considered protective against the sexual health risks to which young people are exposed. There is, however, limited information on the preferred choices of “*where*”, “*how*” and “*from whom*” young people would like to receive SRH information. We aimed to describe the experience and preferences of young people regarding their SRH education and learning and in particular communication with their parents/guardians.

**Methods:**

We conducted a cross-sectional study among randomly selected students aged 18-24y attending Higher Learning Institutions (HLIs) in Mbeya, Tanzania. We used a self-administered questionnaire to collect information on SRH education received, ability to discuss SRH matters with a parent/guardian and SRH information gaps encountered during their early sexual experience.

**Results:**

We enrolled 504 students from 5 HLIs, of whom 446 (88.5%) reported to be sexually active, with mean age at sexual debut of 18.4y (SD 2.2). About 61% (307/504) of the participants found it difficult to discuss or did not discuss SRH matters with their parent/guardian while growing up. Learning about SRH matters was reported from peers (30.2%) and teacher-led school curriculum (22.7%). There was a strong gender-biased preference on SRH matters’ discussions, female and male participants preferred discussions with adults of their respective sex. Peers (18.2%), media (16.2%) and schools (14.2%) were described as the preferred sources of SRH information. On recalling their first sexual experience, sexually-initiated participants felt they needed to know more about sexual feelings, emotions and relationships (28.8%), safer sex (13.5%), how to be able to say *‘No’* (10.7%) and how to use a condom correctly (10.2%).

**Conclusion:**

Young people have a gender preference when it comes to learning about SRH matters from their parents; however, such conversations seldom occur. Community health education should focus on building skills of parents on parent-child communication on SRH matters so as to empower them to confidently initiate and convey accurate SRH information. Comprehensive SRH education and skills building need to be strengthened in the current school SRH curriculum in order to meet the demand and needs of students and increase the competence of teachers.

**Supplementary Information:**

The online version contains supplementary material available at 10.1186/s12889-021-11728-2.

## Introduction

Sexual and Reproductive Health (SRH) among young adults in developing countries is still a major public health challenge [1]. The World Health Organization (WHO) estimates that one in 20 adolescents contract a sexually transmitted infection (STI) each year [2]. Worldwide, almost half of new HIV infections occur in the 15-24y age-group, identifying this group as high-risk; and in Sub-Saharan Africa (SSA), one in six adolescent deaths are attributed to HIV [3]. In Tanzania, 45% of new HIV infection cases are reported among young adults, with less than half of the population aged 15–24 years being accurately informed on HIV/AIDS [4, 5]. Despite mass-media information campaigns on STIs and HIV/AIDS, a number of studies of sexual practices among young adults in low and middle-income countries (LMICs) still show suboptimal levels of the necessary preventive behavior against HIV, STIs, unwanted pregnancies and injuries [6–8]. Consequences of risky sexual activity among young adults include unwanted pregnancies, unsafe abortions, risk for STIs which may be asymptomatic with long-term sequelae, and increased risk of getting HIV [2].

Health-related behaviors, such as sexual activity, are determined by different players within a defined institution; and the key players define meanings and level of acceptance as to the how, who and when [9]. Such meanings then influence decision-making among individuals, e.g. young adults, on matters of sexual behaviors and health care seeking and practices. Sexual behavior is thus an outcome of a variety of factors varying from personal, peer, parents, institutional, societal and even public policies. Early school-based sexuality education programs and sexual health information sharing between teachers and young people have been considered protective against the sexual health risks to which young people are exposed [10, 11]. In Tanzania, schools are considered important frameworks linking young people and their communities, preparing them during the transition to adulthood; and young people look up to their teachers as a preferred, reliable and integral point when it comes to learning about SRH matters [12, 13]. An evaluation of sex and HIV education programs in high-income and LMIC among youth in schools and community settings, found such programs being more likely to influence a positive impact on delaying sex, reducing number of sexual partners, increase in condom and contraceptive use, as well as composite measures of sexual risk reduction [10]. In Tanzania, a number of programs targeting in and out-of-school adolescents and young people have proved beneficial in improving SRH knowledge among young people, but have been less effective in improving safer sexual behaviours and SRH outcomes [14–16].

Parents constitute another group that have a key role in informing and educating adolescents and youths on matters pertaining to SRH, in addition to their responsibility of assuring optimal growth and developmental well-being of their children. Similar to school-based sexuality education programs, parent-child communication can encourage preventive behaviors against risky sexual behaviors among young people [17–19]. Parents can impart moral and cultural values, positive beliefs and skills in order to empower adolescents to make correct decisions and reduce their risk to SRH threats. Studies have shown that young people also prefer parent-child communication with regard to SRH matters but such important conversations do not happen in many settings in SSA [19–21].

In Tanzania, studies have noted that the school SRH curriculum has many shortcomings, lacks resources and that students reported it was an inadequate source of information on SRH matters and opt for other sources (peers, media, and parents/relatives) [22]. The curriculum faces a number of limitations, such as the students’ age when commencing the curriculum in relation to the reported age at sexual debut and ineffective inclusion of sensitive sexuality matters [23, 24]. Young people reported curriculum gaps on sexual decision-making, sexual pleasure, relationships, safer sex and condom use, and masturbation [24]. Young people are thought to receive information on sexual matters from informal sources such as their peers or social media; and their will to take preventive actions may be challenged by barriers or modifying factors towards prevention or treatment of the disease. Misconceptions about level of perception to sexual health risks may result from ignorance as a result of lack of comprehensive sexual education from an early age (example secondary or high school). Gender and sexuality attitudes and values are believed to start being established early in childhood and through adolescence, and eventually dictate sexual behavior and continue to affect the individual through adulthood.

Lack of proper SRH education reflects problems facing young people such as unprotected sex, unplanned pregnancies with unsafe abortions, HIV/STIs [1, 2]. Additional to school-based sexuality education programs and parent-child communication, other avenues for learning may include information sharing with peers, media, newspapers, Internet, and/or health personnel and facilities. In Tanzania, there is limited information on preferred choices of “what”, “*where*”, “*how*” and “*with whom*” young people like to get their SRH information. We aimed to describe the experience and preferences of young people regarding their SRH education and learning, and in particular the importance of communication with their parents/guardians regarding sexuality.

## Methodology

### Study design, settings and population

We conducted a cross-sectional study of students aged 18-24y attending Higher Learning Institutions (HLIs) in Mbeya region, in Southern Tanzania. The region has 6 HLIs registered by the Tanzania Commission for Universities, namely Mbeya University of Science and Technology (MUST), Mzumbe University –Mbeya University College (MUMCo), Tanzania Institute of Accountancy (TIA), Mbeya Teofilo Kisanji University (TEKU), Open University of Tanzania (OUT) and St. Augustine University of Tanzania (SAUT).

Each HLI was invited to participate if they included students aged 18-24y, and thus one institution (Open University of Tanzania, with only older students) was excluded. All other HLIs agreed to take part. A complete electronic list of all registered students of Tanzanian nationality, aged 18-24y, irrespective of year of study was obtained from the Academic Registrars’ offices. Students attending short-term courses (< 6 months) or elective students were not eligible.

Proportions assessment in cross-sectional studies using random sampling was used to estimate sample size adjusted for non-response, and the minimum total sample required was 494. Probability proportional to size was used to determine the total number of students by sex from each HLI according to HLI size (number of registered students). A computerized random number was used to select students irrespective of the course they were registered for.

Each selected eligible student was notified via phone that s/he has been randomly selected to take part in the study and if s/he was willing to take part, s/he was then requested to report to the data collection point within their respective campuses. If the phone number was not reachable or the selected student had no mobile phone, the class representative assisted to physically find the selected student and a face to face appointment was scheduled. Each selected student was required to present a student identification proof and provide written informed consent prior to all study-related procedures.

### Data collection methods, tools and study procedures

The study team applied an individual self-administered questionnaire using a tablet or smart phone through a web-based software (ODK) or hard copy, which ever method the enrolled participant preferred. The questionnaire was customised from various research studies and survey questionnaires to assess sexual risk behaviors (https://www.natsal.ac.uk/media/2097/final-questionnaire_technical-report-appendix-b.pdf) to relate to the Tanzanian setting (Supplementary file 1). Pre-testing of the questionnaire was done on few HLI students from a nearby region prior to data collection; and any emerging issues from the pre-testing were factored in to revise the questionnaire in a more practical manner. The questionnaire was divided into sections, and collected information on socio-demographics (age, religion, marital status, year of study at HLI, highest academic level before current HLI, type of secondary school attended, permanent residence and source of financial support); SRH (ability to discuss sexual matters with a parent/guardian, learning about sexual matters and SRH issues participants felt they ought to have known more about at time of sexual debut or when first felt ready to have some sexual experience). Parent-adolescent communication and preference of source of SRH information was assessed using a set of 10 questions. We referred to sexual matters as sexual activities, sexual identity, sexual interests, sexual orientation, sexuality, sex and sexual relationships.

### Data management and statistical analysis

The web-based software open data kit (ODK) was used to design the questionnaire with smart checks for incomplete or ambiguous responses, and responses through the hard copy questionnaire were reviewed for completeness at the end of each day by a Research Assistant. Data was cleaned and analysed using statistical software Stata version 14 for Windows (Statacorp, College Station, TX 77845, USA). Data was summarised descriptively using percentages and/or proportions for categorical variables, mean and respective measure of dispersion for numerical variables.

### Definition of variables

Knowledge on STIs was assessed by a set of 33 questions and each correct response was given a single mark. Knowledge on STIs was categorized to Good, Moderate or Poor based on correct response above 75, 45–75% and below 45%, respectively.

### Ethics

Complete study information was given to all eligible participants and the ones who agreed to participate, signed an informed consent form prior to any study related activity. Ethical approval was granted by the Mbeya Medical Research and Ethics Review Committee (*Reference Number SZEC-2439/R.A/V.1/07*), Kilimanjaro Christian Medical College Research Ethics and Review Committee (*Reference Number 2405*), and National Health Research Ethics Committee (*Reference Number NIMR/HQ/R.8a/Vol.IX/3092*). Only participant’s study identification number was used in the questionnaire; and only the Principal Investigator and Research Assistant had access to information linking the study identification number and participant’s identifier.

## Results

The study was conducted between March 2019 and January 2020. A total of 632 students aged 18-24y attending 5 HLIs in Mbeya were randomly selected from the HLI registries and contacted by the study team, of whom 504 agreed to participate. Of the 128 who were not enrolled, 32 (25%) could not be reached and the remaining were not willing to participate in the study. No further information was collected following refusal to participate.

### Characteristics of the participants

Participants’ socio-demographic characteristics are shown in Table 1. The mean age of the 504 students was 21.5y (SD 1.7), with more than half of the participants being males (57.3%); the vast majority were single (93.8%) and supported financially by a parent/guardian (86.3%). The majority of participants (78.0%) were in their first or second year of study.

**Table 1:** Socio-demographic characteristics of the participants (*N* = 504)

Variable	Females (***N*** = 215) n (%)	Males (***N*** = 289) n (%)	Total n (%)
Age, years			
18–20	82 (38.1)	70 (24.2)	152 (30.2)
21–24	133 (61.9)	219 (75.8)	352 (69.8)
Mean (SD) age of participants	21.0 (1.8)	21.8 (1.6)	21.5 (1.7)
Religion			
Christian	184 (85.6)	243 (84.1)	427 (84.7)
Muslim	27 (12.5)	36 (12.5)	63 (12.5)
Not reported	4 (1.9)	10 (3.4)	14 (2.8)
Marital status			
Single	194 (90.2)	279 (96.5)	473 (93.8)
Married	8 (3.7)	4 (1.4)	12 (2.4)
In a relationship/cohabiting	13 (6.1)	6 (2.1)	19 (3.8)
University year			
First	109 (50.7)	114 (39.4)	223 (44.3)
Second	70 (32.6)	100 (34.6)	170 (33.7)
Third	33 (15.3)	75 (26.0)	108 (21.4)
Fourth	3 (1.4)	0 (0.0)	3 (0.6)
Type of secondary school attended			
Boarding school	106 (49.3)	136 (47.1)	242 (48.0)
Day school	70 (32.6)	103 (35.6)	173 (34.3)
Both	39 (18.1)	50 (17.3)	89 (17.7)
Financial support			
Parent/Guardian	191 (88.8)	244 (84.4)	435 (86.3)
Sponsorship/Well-wisher	14 (6.5)	23 (8.0)	37 (7.3)
Self	9 (4.2)	21 (7.3)	30 (6.0)
Others	1 (0.5)	1 (0.3)	2 (0.4)
Resident of Mbeya			
Yes	67 (31.2)	84 (29.1)	151 (30.0)
No	147 (68.4)	205 (70.9)	352 (69.8)
Not reported	1 (0.4)	0 (0.0)	1 (0.2)

### Learning about sexual and reproductive health (SRH) matters

Over 70% of the participants lived with both parents while growing up between the ages of 12-18y (Table 2). A high proportion (61%) of participants found it difficult to discuss or did not discuss SRH matters with a parent/guardian during that period, irrespective of participant sex. Two of the most common source of SRH learning during adolescence was from peers (30.2%) and during school lessons (22.7%). Female participants (47.5%) preferred discussing SRH matters with female adults while male participants (42.9%) preferred male adults. SRH education during primary school, secondary school (O′ and A’ level) and at University was reported by 13.3, 90 and 52.8% of the participants, respectively (Table 2).

**Table 2:** Participant’s learning preference and source of information about Sexual and Reproductive Health matters (*N* = 504)

Variable	Females (***N*** = 215) n (%)	Males (***N*** = 289) n (%)	Total (***N*** = 504) n (%)
Parent(s)/guardian(s) living with participant at age 12–18 years			
Both parents	154 (71.6)	221 (76.5)	375 (74.4)
Single parent	31 (14.4)	39 (13.5)	70 (13.9)
Grandparents	17 (7.9)	19 (6.6)	36 (7.1)
Adoptive parents/guardian	13 (6.1)	10 (3.4)	23 (4.6)
Participant’s ability to discuss SRH matters with parent(s)/guardian(s) at age 12–18 years			
Easy (with one or both)	61 (28.4)	79 (27.3)	140 (27.8)
Difficult	72 (33.5)	92 (31.8)	164 (32.5)
Did not discuss (with either)	57 (26.5)	86 (29.8)	143 (28.4)
Varied/depended on topic	25 (11.6)	32 (11.1)	57 (11.3)
How participant learnt about SRH matters while growing up (*multiple responses apply*)			
Father (including step or adoptive)	9 (2.9)	33 (7.8)	42 (5.7)
Mother (including step or adoptive)	41 (13.3)	32 (7.6)	73 (10.0)
Brother(s)/sister(s)	16 (5.2)	38 (9.0)	54 (7.4)
Friends of about my own age	93 (30.1)	128 (30.3)	221 (30.2)
Lessons at school	79 (25.6)	87 (20.6)	166 (22.7)
Media/Books/newspapers/Internet/pornographic websites	42 (13.6)	75 (17.7)	117 (16.0)
Doctor, nurse or clinic	12 (3.9)	11 (2.6)	23 (3.1)
First (girlfriend/boyfriend) or sexual partner	14 (4.5)	18 (4.3)	32 (4.4)
Others	3 (1.0)	1 (0.2)	4 (0.5)
Preferred parent/guardian sex when discussing sexual matters			
Male adult only	7 (3.2)	124 (42.9)	131 (26.0)
Female adult only	103 (47.9)	34 (11.8)	137 (27.2)
I had no preference	96 (44.7)	114 (39.4)	210 (41.6)
Not reported	9 (4.2)	17 (5.9)	26 (5.2)
Participant received Sexual and Reproductive Health Education (SRE) while at school			
Yes	160 (74.4)	225 (77.9)	385 (76.4)
No	55 (25.6)	64 (22.1)	119 (23.6)
Level at which SRH education received *(multiple responses apply)*			
Primary school	27 (12.1)	40 (12.5)	67 (13.3)
Secondary school O′ level	142 (63.7)	211 (65.7)	353 (70.0)
Secondary school A’ level	43 (19.3)	58 (18.1)	101 (20.0)
Others	11 (4.9)	12 (3.7)	23 (4.6)
Ever received a SRH education while at University			
Yes	108 (49.8)	158 (55.1)	266 (52.8)
No	109 (50.2)	129 (45.0)	238 (47.2)

### Sexual and reproductive health information gaps around time of sexual debut

On recalling their first sexual experience, sexually-initiated participants (446/504, 88.5%) reported initiating sex because they were in love (35%), got carried away (24.9%) and were just curious (15.5%)(Table 3).

**Table 3:** Sexual and Reproductive Health information gaps during early sexual experience (*N* = 446)

Variable	Females (***N*** = 185) n (%)	Males (***N*** = 261) n (%)	Total n (%)
Age at sexual debut, years			
< 15	5 (2.7)	39 (15.0)	44 (9.9)
16–18	65 (35.1)	100 (38.3)	165 (37.0)
> 18	9 (51.4)	100 (38.3)	195 (43.7)
Not reported	20 (10.8)	22 (8.4)	42 (9.4)
Mean (SD) age of sexual debut	19 (1.8)	17.9 (2.4)	18.4 (2.2)
Reasons for having sex			
I was curious	21 (11.4)	48 (18.4)	69 (15.5)
I was carried away	39 (21.1)	72 (27.6)	111 (24.9)
Most people in my age group doing it	10 (5.4)	35 (13.4)	45 (10.1)
It seemed like a natural *‘follow on’* in the relationship	6 (3.2)	10 (3.8)	16 (3.6)
I was in love	76 (41.1)	80 (30.7)	156 (35.0)
I wanted to lose my virginity	3 (1.6)	3 (1.2)	6 (1.4)
Other	25 (13.5)	8 (3.0)	33 (7.3)
Not reported	5 (2.7)	5 (1.9)	10 (2.2)
How, or from whom, would you have liked to learn about SRH matters that you were not conversant about during early sexual experience (*SRH information gap from* Fig. 1) *(multiple responses apply)*			
Father (including step or adoptive)	17 (3.2)	84 (11.0)	101 (7.8)
Mother (including step or adoptive)	118 (22.4)	43 (5.6)	161 (12.5)
Brother(s)/sister(s)	20 (3.8)	106 (13.8)	126 (9.7)
Friends of about my own age	87 (16.5)	148 19.3)	235 (18.2)
First (girlfriend/boyfriend) or sexual partner	48 (9.1)	53 (6.9)	101 (7.8)
Lessons at school	63 (12.1)	120 (15.6)	183 (14.2)
Media/Books/newspapers/Internet/pornographic websites	80 (15.2)	130 (16.9)	210 (16.2)
Doctor, nurse or clinic	88 (16.7)	79 (10.3)	167 (12.9)
Others	5 (1.0)	4 (0.5)	9 (0.7)
If diagnosed with an STI, who will you tell *(multiple responses apply)*			
Friends of about my own age	29 (10.9)	95 (21.4)	124 (17.5)
Family member (Mother/Father)	87 (32.7)	134 (30.2)	221 (31.2)
Girlfriend/Boyfriend or sexual partner	111 (41.7)	116 (26.2)	227 (32.0)
Brother/Sister	25 (9.4)	80 (18.1)	105 (14.8)
No one	14 (5.3)	18 (4.1)	32 (4.5)

When asked from whom they would have liked to learn more on SRH matters that they knew little about at time of sexual debut, 20.3% would have preferred parents (combined, father – 7.8% and mother – 12.5%), 18.2% would have liked to learn from friends, 16.2% wanted to learn from media/books/newspapers/Internet/pornographic websites and 14.2% from lessons at school (Table 3). If diagnosed with an STI, participants would rather tell a sexual partner (32%) or family member (31.2%) than other people.

**Fig. 1:**
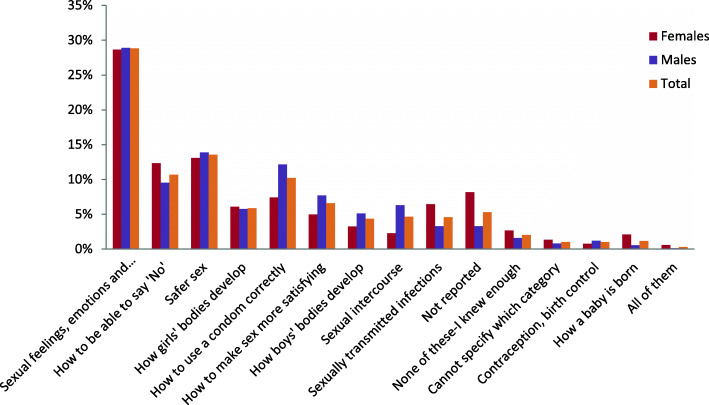
SRH information that participants would have liked to know more about at time of sexual debut or when first felt ready to have a sexual experience (*N* = 446)

Figure 1 shows what participants would have liked to know more about at time of sexual debut or when first felt ready to have a sexual experience: 28.8% said they wished they knew about sexual feelings, emotions and relationships, 13.5% about safer sex, 10.7% about how to be able to say ‘No’ and 10.2% about how to use a condom correctly.

## Discussion

Young people often start discovering and learning primarily about sexual issues from their peers and/or sexual partners, as reported in our findings. In the process of growing up, there is a flexible shift of emotional bonds from parents/guardians to peers to sexual partners [25]. Times when these emotional bonds peak are also likely to be convenient learning times on SRH matters; and parents often have the initial and most crucial stage and are at an added advantage. Adolescence is a time when the family can not quite define their young ones as children or yet adults [25], and this is a period when the habitual position of the parents/guardians may get overridden slowly by peers/friends of a similar age. Parents/guardians may face challenges when it comes to speaking to young adults on SRH matters as they may lack sufficient knowledge [26] or some may believe that sharing SRH information will encourage sexual practices among their children [27]. In situation where parents/guardians do communicate about SRH matters, then talks or conversations have been known to either be threatening, authoritative, subjective to an already existing incidence [26, 28], triggered through examples of acquaintances who suffered STIs/HIV or messages from radio/TV programs [20]. In a qualitative exploration of sexual health by Kajula et al., parents were likely to use fear to discourage unaccepted sexual behaviors among their children and children noted that sexual health conversations with parents were unclear and just full of warnings about the dangers of STIs/HIV [20].

Traditionally, many African communities would have a scheduled time for adolescents to meet with community or family elders (i.e. respectable distinguished men and women) who would take them through information on SRH matters and which would generally tend to emphasize appropriate behaviors. More recently, and in part caused by urbanization and lifestyle changes, many aspects of such cultures have disintegrated [29] and the family structure is left to operate on its own. It is therefore important for parents/guardians of adolescents to be able to speak up calmly, clearly and without fear or shame about sexuality and SRH matters with their children in a friendly, positive way. Parent-child communication can actively promote desirable preventive behaviors against sexual health threats among young people. As with other developmental and behavioral aspects which parents/guardians can advocate among their children, sexual behavior and attitudes towards sexual health can also be learnt and influenced at the household/family level. Evening or weekend radio/TV programs could usefully be used to set off such family talks as in most urban and some rural African settings, parents/guardians after work would sit with their children to unwind while watching news or comedy shows.

Adolescents and young adults would prefer to talk to their parents/guardians as evident from our findings and those reported elsewhere [20, 21, 28]*,* although they would prefer to talk to someone of the same sex, as noted elsewhere [21, 26, 30]. Parents/guardians need to recognize and accept that it is also their responsibility to protect their children against potential sexual health risks, and that responsibility should not entirely be delegated to teachers, health care workers or their children’s peers. Programs advocating parent-child communication and SRH among adolescents/young adults need to also focus on strengthening parental communication skills regarding sexuality matters, self-efficacy as well as appropriate SRH knowledge. Unsatisfactory methods of parent-child communication about SRH matters are confusing and rarely successful in modifying sexual behaviors of adolescents and young adults who are highly experimental and fragile.

A little over three-quarters of study participants reported to have received a SRH education while at school and, for the majority, during their secondary education. However, participants felt they needed to know more about sexual feelings, emotions and relationships, safer sex, how to be able to say *‘No’* and how to use a condom correctly at the time of sexual debut. Introduction of SRH education earlier on in life carry considerable benefits for delaying sexual debut, addressing misconceptions, preventing risky sexual behaviors and eventually STIs/HIV and/or pregnancy [2, 31]. School-based SRH education has been found effective in preventing STIs and is highly recommended worldwide [32, 33]; and teachers through SRH education given in schools, play a vital role in informing sexual behavior among young people that helps prevent STIs/HIV [16, 32, 34]. However, evaluations of SRH education curricula in sub-Saharan Africa have been inconclusive on their impact in reducing risky sexual behaviors and the incidence of STIs/HIV among young adults [32, 35]. The SRH education curriculum that was introduced in Tanzania in primary schools during the early 2000’s to help with the control of the HIV pandemic has been important for educating adolescents in primary and secondary schools, but its main challenge has been the inability of many teachers to deliver the message and topics clearly [36]. Whilst Mkumbo et al. showed that the majority of urban and rural teachers in Tanzania were supportive of SRH education teaching [13], Cardoso and Mwolo noted an ineffective inclusion of sensitive sexuality matters in the school curricula and inadequate resources [36].

In this study, peers, lessons at schools and media were identified as key sources of SRH information and sex education. However, participants who claim to have received the SRH education and have already experienced sexual encounters needed to know more about sexual feelings, emotions, relationships, safer sex practices and ability to turn down a sexual encounter. It is crucial to understand whether the SRH education curricula indeed cover the “sexual” aspect. Could it be that the “*sexual*” aspect is missing from the SRH education? Could it be that teachers are still not comfortable to teach such sensitive issues considering our culture that may label such topics as taboo? Nearly a decade after Mkumbo’s work on teachers’ attitudes towards and comfort about teaching school-based SRH education in urban and rural Tanzania [13], it is probable that, even though teachers would have liked to teach, they experience difficult and discomfort in teaching most of the key sexuality education topics. It is important to send across clear messages to adolescents from a young age and throughout secondary and higher learning on sensitive SRH matters, also to build capacity of teachers with the correct and up-to-date knowledge as well as the confidence to teach. As peers are the typical source of information on SRH matters among adolescents and young adults, it is unclear how well-informed they are and how reliable is the information shared. Sexual practices and behaviors being largely influenced by peers is directly related to the circle of information one is in. It is therefore important to invest on availability of comprehensive SRH information among adolescents and young adults within schools and their surrounding communities so as to make the information they share credible.

Nearly half of the participants reported receiving SRH education while at University. In Tanzania, implementation of health education and awareness campaigns in HLIs are compromised either due to lack of policies and staff commitment or financial prospects [37]. Funding for such activities is mainly from external sources and often SRH education programs are not streamlined [37]. At HLIs, there have been reports on a dearth of SRH initiatives and if present, there are concerns about their quality and appeal. Health education campaigns have been perceived as “*boring*”, “*repetitive*” or “*normal*”; and senior staff members have been perceived to lack commitment to SRH matters [37]. Students in HLIs are generally young adults within the defined STI/HIV high-risk group of 15–24 year olds, probably at their peak years of sexual activity, curiosity and experimentation; and many are free of immediate parental supervision. Studies have shown that while the majority of students believe that they are at low or no risk to STI/HIV, they were actually found to be at high risk after assessing their sexual behavior practices [6]. Regional and district health management teams could be engaged to assist HLIs with regard to the health promotion components of SRH education and involved in targeted health awareness campaigns to address students’ sexual health risks.

Our study findings may have been limited by recall bias from recalling past experiences, especially those of a sexual nature which were very personal and sensitive. Also, we only addressed a limited number of family factors and did not probe in depth some other family factors that may influence parent-child communication. The level of SRH education received by participants was not uniform as some had received SRH education starting from primary school level and others only later. Since quite a significant number of adolescents do not gain post-primary/secondary education in Tanzania, our study findings may not be generalizable to those who have not attained higher learning education.

## Conclusion

Young people have a gender preference when it comes to learning about SRH matters from their parents; however, such conversations seldom occur. Sexual behavior among adolescents and young people is influenced largely by familial, structural and environmental factors; and therefore, talks on SRH matters need to actively begin at the family level. Community health information, education and communication (IEC) should focus on building skills of parents on parent-child communication on SRH matters so as to empower them to confidently initiate and convey accurate SRH information. Further, comprehensive SRH education and skills building need to be strengthened in the current school SRH curriculum in order need to meet the demand and needs of students and increase the competence and confidence of teachers.

## Supplementary Information

**Additional file 1: Supplementary file 1.** Individual self-administered questionnaire, customised from various research studies and survey questionnaires to assess sexual risk behaviors among adolescents and young adults to relate to the Tanzanian setting.

## Data Availability

The datasets generated and analysed during the current study are available at Figshare: Where and how do young people like to get their Sexual and Reproductive health information? 10.6084/m9.figshare.13049951 This study contains the following underlying data: • Data file 1 (Dataset) • Data file 2 (Dataset variables key)

## Abbreviations

HLIs: High Learning Institutions
IEC: Information, Education and Communication
LMICs: Low and Middle-Income Countries
MUMCo: Mzumbe University Mbeya University College
MUST: Mbeya University of Science and Technology
ODK: Open Data Kit
OUT: Open University of Tanzania
SAUT: St. Augustine University of Tanzania
SRH: Sexual and Reproductive Health
SSA: Sub-Saharan Africa
STI: Sexually Transmitted Infection
TCU: Tanzania Commission for Universities
TEKU: Teofilo Kisanji University (Mbeya)
TIA: Tanzania Institute of Accountancy
WHO: World Health Organization
