# Supplementary material for: Where and how do young people like to get their sexual and reproductive health (SRH) information? Experiences from students in higher learning institutions in Mbeya, Tanzania: a cross-sectional study
Source: BMC Public Health. 2021 Sep 16;21:1683. doi: 10.1186/s12889-021-11728-2 (PMC8444543; doi:10.1186/s12889-021-11728-2)
Supplement: Supplementary file 1 — Additional file 1: Supplementary file 1. Individual self-administered questionnaire, customised from various research studies and survey questionnaires to assess sexual risk behaviors among adolescents and young adults to relate to the Tanzanian setting. [file 12889_2021_11728_MOESM1_ESM.pdf]

LONDON SCHOOL of HYGIENE &TROPICAL MEDICINE

NIMR - MMRC

Appendix B1: Self-administered Questionnaire

Sexually Transmitted Infections (STIs) and sexual behaviour among young adults attending Higher Learning Institutions (HLIs) in Mbeya region, Tanzania.
Protocol Version: 1.1, dated 01 Dec 2018.

In this study, the Investigators would like to understand the prevalence of HIV, other potential STIs and sexual behaviour among young adults enrolled in Higher Learning Institutions (HLIs) in Mbeya region in different years of study. We would ask you to respond to the following questions which are important to find out about sexual behaviour practices and level of awareness to STIs. Please be as open as possible. All information provided herein will be kept strictly confidential and for the study purpose will not be linked to your name.

A. SOCIO-DEMOGRAPHICS

1. Do you identify yourself as **O** Female **O** Male
2. Age: ____________________
3. Tribe: ___________________
4. Religion: ________________
5. Marital status: **O** Single **O** Married **O** Cohabiting **O** Divorced/Separated
   O Others (please mention) ……………………………………….
   5.1 If married; age at first marriage _________________
6. Year of Uni: **O** First **O** Second **O** Third **O** Fourth
7. Highest academic level before the current University ………………………………..
8. Employment status: **O** Employed **O** Un-employed
9. Source of financial support: **O** Self **O** Parent/Guardian **O** Sponsorship/Well-wisher
   O Others (please mention) ………………………………
10. Permanent Residence (Region): ______________________

B. SEXUAL HEALTH EDUCATION

11. During your secondary education, which type of school did you attend?
    O 1. A boarding school
    O 2. A day school
    O 3. Both, a day and a boarding school
    O 4. Other (please mention) …………………………………

12. Who were you living with when you were aged 12-18? (Stepfather/Stepmother includes cohabiting partners of natural parent). If you have multiple answers, consider the one you lived with the longest.
    O 1. Natural mother and father

O 2. Mother and stepfather
O 3. Father and stepmother
O 4. Mother only
O 5. Father only
O 6. Grand parents
O 7. Adoptive mother and father
O 8. In Local Authority care / Foster home
O 9. Other (please say who) …………………………………

13. When you were 12-18, did you find it easy or difficult to talk to your (answer from previous question) about sexual matters, or didn't you discuss sexual matters with (them/her/him) at that age?
O 1. Easy (with one or both)
O 2. Difficult
O 3. Didn't discuss (with either)
O 4. Varied/depended on topic

14. If you did discuss sexual matters (answer from previous question), which gender were you more comfortable discussing sexual matters with?
O 1. Male adult only
O 2. Female adult only
O 3. I had no preference

15. When you were growing up, in which of the following ways did you learn about sexual matters? (Rank your responses with consecutive numbers - 1 being the one you learnt the most)
O 1. Father (including step or adoptive)
O 2. Mother (including step or adoptive)
O 3. Brother(s) / sister(s) (including half, step, or adoptive)
O 4. Friends of about my own age
O 5. First (girlfriend/boyfriend) or sexual partner
O 6. Lessons at school
O 7. Television / radio / DVDs / videos
O 8. Books / magazines / newspapers
O 9. Internet/pornographic websites/Pornographic magazines / films
O 10. Doctor, nurse or clinic
O 11. Other (Please mention) ……………………………………. …………..

16. Have you ever received a sex and relationships education (SRE) class while at school?
O Yes O No

17. If Yes, at which level? (select all that apply)
O 1. Primary school
O 2. Secondary school O' Level

O 3. Secondary school A' Level
O 4. Other (Please mention) ……………………………….

18. Have you ever received a sex and relationships education (SRE) class while in University?
O Yes **O** No

C. KNOWLEDGE AND ATTITUDE ON STIs

19. Have you ever heard of Sexually Transmitted Infections (STIs)? **O** Yes **O** No

20. How did you get to know about STIs? (Rank your responses with consecutive numbers - 1 being the one you learnt the most)
O 1. Father (including step or adoptive)
O 2. Mother (including step or adoptive)
O 3. Brother(s) / sister(s) (including half, step, or adoptive)
O 4. Friends of about my own age
O 5. First (girlfriend/boyfriend) or sexual partner
O 6. Lessons at school/University
O 7. Television / radio / DVDs / videos
O 8. Books / magazines / newspapers
O 9. Internet/pornographic websites/Pornographic magazines / films
O 10. Doctor, nurse or clinic
O 11. Other (Please mention) ……………………………………………..

21. Which of the following is an STI? (select all that apply)
O 1. Gonorrhea
O 2. Syphilis
O 3. Genital herpes
O 4. Trichomoniasis
O 5. Tuberculosis
O 6. Asthma
O 7. HIV/AIDS
O 8. Chlamydia
O 9. Hepatitis B
O 10. Hepatitis C
O 11. Human Papilloma Virus
O 12. Bacterial Vaginosis

22. What are the causative organisms of STIs? (select all that apply)
O 1. Bacteria
O 2. Virus
O 3. Fungi
O 4. Parasites
O 5. Mosquitoes

23. What are the routes of transmission of STIs? (select all that apply)
- O 1. Sharing food/drinks
- O 2. Sharing clothes/underwear/towels
- O 3. Sharing injection needles
- O 4. Blood transfusion
- O 5. Infected mother to a child
- O 6. Kissing
- O 7. Penetrative Sexual intercourse
- O 8. Non-Penetrative Sexual intercourse (e.g oral sex)

24. What are the symptoms of STIs? (select all that apply)
- O 1. Ulcers in the genital organs
- O 2. Pain while passing out urine
- O 3. Swollen glands, fever and body ache
- O 4. Discharge from the penis
- O 5. Discharge from the vagina
- O 6. Itching around the vagina
- O 7. Sore throat
- O 8. Painless sores on the mouth
- O 9. Painless sores on genital areas

25. What are the complications of STIs? (select all that apply)
- O 1. Infertility
- O 2. Cervical cancer
- O 3. Death
- O 4. Ectopic pregnancy
- O 5. Still birth
- O 6. Body weakness

For the corresponding statements below, please respond whether you Agree (*Yes*), Disagree (*No*) or *Do not Know*;

**Statement**	**Yes**	**No**	**Do not know**
26. Do contraceptive pills reduce the risk of STIs?	O	O	O
27. Does using condoms decrease the risk of being infected with an STI?	O	O	O
28. Can monogamy reduce one's chance of contracting an STI?	O	O	O
29. Does having multiple sexual partners increase chances of being infected with an STI?	O	O	O
30. Does being infected with one STI increase the risk of infection with another STI?	O	O	O
31. Do you think people infected with one STI can be at an increased risk of getting HIV?	O	O	O
32. Does alcohol intake increase an individual's susceptibility to STIs?	O	O	O
33. Can intake of some illegal drugs increase an individual's susceptibility	O	O	O

to STIs?			
34. Is sexual abstinence the most effective means of avoiding STIs?	O	O	O
35. Can people with STIs have no symptoms?	O	O	O
36. Can you tell if someone or your sexual partner has an STI?	O	O	O
37. If you were diagnosed with an STI, would you inform your sexual partner and allow her/him to also receive treatment?	O	O	O

38. If you were diagnosed with an STI, who will you tell? (Select all that apply)
O 1. Friends of about your own age
O 2. Family member (Mother/ Father, including step, or adoptive)
O 3. Girlfriend/boyfriend or sexual partner
O 4. Brother(s) / sister(s) (including half, step, or adoptive)
O 5. No one
O 6. Other (Please mention) ………………………………….

For the corresponding statements below, please respond whether you *Agree*, *Disagree* or *have No comment*;

**Statement**	**Agree**	**Disagree**	**No comment**
39. If both partners are infected with an STI, I feel there is no need of using a condom	O	O	O
40. I feel it is necessary for academic institutions to discuss issues regarding prevention of STIs	O	O	O
41. I feel banning of prostitution can control the spread of STIs	O	O	O
42. I feel screening for STIs is good	O	O	O
43. I feel screening for STIs before marriage is important	O	O	O
44. I think watching/reading pornographic materials can contribute to risky sexual practices	O	O	O
45. STIs are not dangerous because they can be cured	O	O	O
46. In my opinion, I feel STIs can cause death if left untreated	O	O	O
47. I am worried about contracting STIs	O	O	O
48. The STI problem is something that I have not given much thought to	O	O	O
49. Same sex relationships are solely to be blamed for the spread of STIs	O	O	O
50. If I have unprotected sexual intercourse, I am most concerned about:<br>- Getting HIV<br>- Getting STIs aside from HIV<br>- Unwanted pregnancy<br>- I am not concerned	 O<br>O<br>O<br>O	 O<br>O<br>O<br>O	 O<br>O<br>O<br>O
51. If I notice symptoms of STIs, I think I should seek treatment immediately	O	O	O
52. If I notice symptoms of STIs in my partner, I will advise him/her to seek treatment immediately	O	O	O

D. **SEXUAL ACTIVITY/BEHAVIOUR** (Please remember that your answers are strictly confidential.)

53. How old were you when you first had any type of experience of a sexual kind - for example, kissing, petting, or feeling one another - with someone of the opposite sex?
O 1. Age …………. (please fill in)
O 2. This has never happened

54. How old were you when you first had sexual intercourse with someone of the opposite sex? (Sexual intercourse means a man's penis inside a female vagina).
O 1. Age …………. (please fill in)
O 2. This has never happened

55. The first time you had sexual intercourse with someone of the opposite sex, how old was that partner at that time?
O 1. Age …………. (please fill in)
O 2. This has never happened

56. Who was more willing to have sexual intercourse that first time?
O 1. Both were equally willing
O 2. I was more willing
O 3. Partner was more willing
O 4. I was forced

57. Was any form of contraception used on that occasion? (select all that apply).
O 1. Condom
O 2. Pills / Injectables
O 3. Emergency contraception
O 4. No contraception used
O 5. Other (please mention) ………………………….

58. Which one of these descriptions best applies to you and this person at that time?
O 1. It was someone I didn't know
O 2. We had recently met
O 3. We had known each other for a while, but were not in a steady relationship
O 4. We were in a steady relationship
O 5. We were living together as a couple / married at the time
O 6. Other (please mention) ……………………………………….

59. How did you meet the person who you first had sexual intercourse with?
O 1. Neighbour/lived locally/house or flat-share
O 2. Introduced by friends or family

O 3. At school (primary/secondary)
O 4. At university or college
O 5. At work (or through work)
O 6. Through a sports club, faith group, or other organisation / society
O 7. On holiday or while travelling
O 8. In a pub, bar, night club, dance, or disco
O 9. In a public place (e.g. park, cafe, shop, public transport)
O 10. Internet dating website/social media
O 11. Other (please mention) ……………………………………..

60. Looking back now to the first time you had sexual intercourse, do you think ...
O 1. You should have waited longer before having sex with anyone
O 2. You should not have waited so long
O 3. It was the right time

61. Which of these things applied to you at the time?
O 1. I was curious about what it would be like
O 2. I was carried away by my feelings
O 3. Most people in my age group seemed to be doing it
O 4. It seemed like a natural *'follow on'* in the relationship
O 5. I was in love
O 6. I wanted to lose my virginity
O 7. I was a bit drunk at the time
O 8. I had smoked some cannabis/ used some other drugs
O 9. Other (please mention) …………………………………….

62. Looking back to the time when you first felt ready to have some sexual experience yourself, is there anything on this list that you now feel you ought to have known more about? (select all that apply, if possible rank your responses with consecutive numbers - 1 being the one you prefer the most).
O 1. How to be able to say 'No'
O 2. How girls' bodies develop
O 3. How boys' bodies develop
O 4. Sexual feelings, emotions and relationships
O 5. Sexual intercourse
O 6. Safer sex
O 7. How to use a condom correctly
O 8. How to make sex more satisfying
O 9. Contraception, birth control
O 10. Sexually transmitted infections (e.g. Gonorrhea/ HIV)
O 11. How a baby is born
O 12. Would have liked to know more but can't specify which
O 13. All of them
O 14. None of these - I knew enough

O 15. None - not ready for sexual experience yet

63. How, or from whom, would you have liked to learn more about those sexual matters? (Please select all that apply, rank your responses with consecutive numbers - 1 being the one you prefer the most)
O 1. Father (including step or adoptive)
O 2. Mother (including step or adoptive)
O 3. Brother(s) / sister(s) (including half, step, or adoptive)
O 4. Friends of about my own age
O 5. First (girlfriend/boyfriend) or sexual partner
O 6. Lessons at school/university
O 7. Television / radio / DVDs / videos
O 8. Books / magazines / newspapers
O 9. Internet/pornographic websites/Pornographic magazines / films
O 10. Doctor, nurse or clinic
O 11. Other (Please mention) ……………………………………. ……………..

64. If you are sexually active or have ever been sexually active, how many partners have you had in your lifetime, in total?
Please fill in ………………

65. In the past 6 months, with how many men/women have you had sexual intercourse?
Please fill in ………………

66. In the past 4 weeks, with how many men/women have you had sexual intercourse?
Please fill in ………………

67. Of the persons you had sexual intercourse with in the past 6 months, how many were five or more years older than you?
Please fill in ………………

68. Of the persons you had sexual intercourse with in the past 6 months, how many were five or more years younger than you?
Please fill in …………………. .

69. Of the persons you had sexual intercourse with in the past 4 weeks, how many were five or more years older than you?
Please fill in ………………

70. Of the persons you had sexual intercourse with in the past 4 weeks, how many were five or more years younger than you?
Please fill in …………… .

71. Did you use a condom / Was a condom used when you had sexual intercourse in the last 6 months?

O 1. Yes, used every time
O 2. Yes, used sometimes
O 3. No, not used in the last 6 months

72. Did you use a condom / Was a condom used when you had sexual intercourse in the last 4 weeks?

O 1. Yes, used every time
O 2. Yes, used sometimes
O 3. No, not used in the last 4 weeks

73. How long was it between when you first met your sexual partner and when you first had sex with him/her?

O 1. 24 hours or less
O 2. Between 1 day and 1 week
O 3. Between 1 week and 4 weeks
O 4. Between 4 weeks and 6 months
O 5. Between 6 months and 1 year
O 6. More than 1 year

74. The last time you had sexual intercourse, what method did you or your partner use to prevent pregnancy? (select all that apply).

O 1. I have never had sexual intercourse
O 2. No method was used to prevent pregnancy
O 3. Partner withdrawal
O 4. Condoms
O 5. Birth control pills
O 6. Injectable contraceptive (such as Depo-Provera)
O 7. An intra-uterine device (IUD) or implant (such as Implanon)
O 8. Other (please mention) ……………………………………….

75. As an outcome of a sexual encounter, what do you fear most? (select all that apply).

O 1. Pregnancy
O 2. Contracting an STI
O 3. Contracting HIV
O 4. None of the above

76. Have you ever become pregnant / made someone pregnant?

O Yes **O** No

77. If you answered yes above, how long had you and your partner been sexually active at the time of conception?

Please fill in …………….

78. What decision did you and/or your partner make regarding the pregnancy?
O 1. It was a miscarriage
O 2. We terminated/aborted the pregnancy
O 3. It was a stillbirth
O 4. It was a live birth

79. If you thought that you might have an infection that is transmitted through sex, where would you first go to seek diagnosis and/or treatment?
O 1. Father (including step or adoptive)
O 2. Mother (including step or adoptive)
O 3. Brother(s) / sister(s) (including half, step, or adoptive)
O 4. Friends of about my own age
O 5. Sexual partner
O 6. Pharmacy
O 7. Private clinic or Doctor/nurse
O 8. General hospital/Reproductive health clinic
O 9. Internet
O 10. Other (Please mention) …………………………………….

80. Have you ever been tested for STIs?
O Yes O No

81. Have you ever had a test for HIV?
O Yes O No

82. Have you ever been told by a doctor or other healthcare professional that you had an STI?
O Yes O No

83. Have you ever been told by a doctor or other healthcare professional that you had any of the following?
O 1. Chlamydia
O 2. Gonorrhoea
O 3. Genital Warts
O 4. Syphilis
O 5. Trichomonas vaginalis (Trich, TV)
O 6. Herpes (genital herpes)
O 7. Hepatitis B
O 8. HIV
O 9. Pelvic Inflammatory Disease (PID, salpingitis)
O 10. Vaginal thrush (Candida, Yeast infection)
O 11. Bacterial vaginosis
O 12. Yes, but can't remember which
O 13. None of these

84. When you were last tested for STI, where were you offered the test?

O 1. I was offered the test kit by a friend
O 2. I was offered the test kit by my sexual partner
O 3. Pharmacy / chemist
O 4. Private clinic or doctor
O 5. School / college / university clinic or dispensary
O 6. General hospital/ Reproductive health clinic
O 7. This has never happened

85. Why were you last tested for STI?

O 1. I had symptoms
O 2. My partner had symptoms
O 3. I was notified because a partner was diagnosed with an STI
O 4. I wanted a general sexual health check-up
O 5. Check up after previous positive test
O 6. I had no symptoms but I was worried about the risk of an STI
O 7. I was offered a routine test
O 8. Other (please mention) ……………………………………………….

86. In the last month, have you had any of the following symptoms? (select all that apply).

O 1. Pain, burning or stinging when passing urine
O 2. Passing urine more often than usual
O 3. Genital wart / lump
O 4. Genital ulcer / sore
O 5. Abnormal vaginal discharge/ with or without itching
O 6. Unpleasant odour associated with vaginal discharge
O 7. Vaginal pain during sex
O 8. Abnormal bleeding between periods
O 9. Bleeding after sex (not during a period)
O 10. Lower abdominal or pelvic pain (not related to periods)
O 11. None of these

87. Do you know if your current partner has been tested for STIs?

O Yes O Do not know

88. Do you know if your current partner has tested for HIV?

O Yes O Do not know

89. How old were you when you had your first drink of alcohol?

O 1. Age …………. (please fill in)
O 2. This has never happened

90. Have you ever had sexual intercourse while under the influence of alcohol?

O Yes O No

91. Do you drink alcohol before having sexual intercourse?
O Yes **O** No

92. How old were you when you tried marijuana/other illegal drugs for the first time?
O 1. Age ………… . (please fill in)
O 2. This has never happened

93. Have you ever had sexual intercourse while under the influence of marijuana/other illegal drugs?
O Yes **O** No

94. Do you take marijuana/other illegal drugs before having sexual intercourse?
O Yes **O** No

95. Do you read/watch pornographic materials?
O Yes **O** No

96. Have you ever performed oral sex on your sexual partner? (This means putting your mouth on a penis, vagina or genitals).
O Yes **O** No

97. How old were you when you first performed oral sex on a man/woman?
O 1. Age ………… . (please fill in)
O 2. This has never happened

98. Have you ever had oral sex while under the influence of drugs or alcohol?
O Yes **O** No

99. In the past 6 months, on how many men/women have you performed oral sex?
O 1. Please fill in ………… . ..
O 2. This has never happened

100. In the past 4 weeks, on how many men/women have you performed oral sex?
O 1. Please fill in ………… . ..
O 2. This has never happened

101. Have you ever had anal sex? This means penetration between a man's penis and an anus/back passage.
O Yes **O** No

102. How old were you when you first had anal sex?
O 1. Age ………… . (please fill in)
O 2. This has never happened

103. Have you ever had anal sex while under the influence of drugs or alcohol?
O Yes   O No

104. In the past 6 months, with how many men/women have you had anal sex?
O 1. Please fill in …………….
O 2. This has never happened

105. In the past 4 weeks, with how many men/women have you had anal sex?
O 1. Please fill in …………….
O 2. This has never happened

106. Did you use a condom / Was a condom used when you had anal sex?
O Yes   O No

107. In the last 6 months, have you travelled outside Mbeya region, for any reason?
O Yes   O No

108. Have you had sex with anyone for the first time while you were outside Mbeya region?
O Yes   O No

109. In the last 6 months, how many people did you have sex with for the first time while you were outside Mbeya?
O 1. Please fill in …………….
O 2. This has never happened

110. How did you meet the person(s) who you first had sexual intercourse with for the first time while you were outside Mbeya? (select all that apply).
O 1. Neighbour/lived locally/house or flat-share
O 2. Introduced by friends or family
O 3. At school (primary/secondary)
O 4. At university or college
O 5. At work (or through work)
O 6. Through a sports club, faith group, or other organisation / society
O 7. On holiday or while travelling
O 8. In a pub, bar, night club, dance, or disco
O 9. In a public place (e.g. park, cafe, shop, public transport)
O 10. Internet dating website/social media
O 11. Other (please mention) ……………………………………..

111. Have you ever been physically forced to have sexual intercourse when you did not want to?
O Yes   O No

112. During the past 6 months, how many times did anyone force you to do sexual things that you did not want to do? (count such things as kissing, touching, or being physically forced to have sexual intercourse.)
O 1. Please fill in ……………..
O 2. This has never happened

113. During the past 6 months, how many times did someone you were dating or going out with force you to do sexual things that you did not want to do? (count such things as kissing, touching, or being physically forced to have sexual intercourse.)
O 1. Please fill in ……………..
O 2. This has never happened

114. Are you aware of the presence of a vaccine against the virus that causes cervical cancer, known as HPV (Human Papilloma Virus)?
O Yes **O** No

115. Have you ever been vaccinated against HPV?
O 1. Yes – I have completed three doses of the vaccine
O 2. Yes - I have had one or two doses of the vaccine, but not all three doses
O 3. No

116. Were you ever offered the vaccination, through any national/ school/ university based programme?
O 1. Yes, I was offered but refused it
O 2. No, I was never offered it

117. Where (were you offered/ did you receive) the HPV vaccination?
O 1. School (Primary/Secondary)
O 2. Private clinic or doctor, including a chemist / pharmacy
O 3. Sexual health clinic
O 4. General hospital
O 5. Somewhere else, please mention ……………………………

118. Have you ever been vaccinated against Hepatitis B?
O Yes **O** No/I do not know

119. Do you think of yourself as . . . (select a single response)
O 1. Heterosexual (attracted to opposite sex only)
O 2. Homosexual (attracted to same sex only)
O 3. Bisexual (attracted to men and women)
O 4. Prefer not to say

THANK YOU FOR COMPLETING THIS QUESTIONNAIRE.
